# Therapeutic Implications of Renin–Angiotensin System Modulators in Alzheimer’s Dementia

**DOI:** 10.3390/pharmaceutics15092290

**Published:** 2023-09-06

**Authors:** Daniela-Carmen Ababei, Veronica Bild, Ioana Macadan, Alexandru Vasincu, Răzvan-Nicolae Rusu, Mihaela Blaj, Gabriela Dumitrița Stanciu, Radu-Marian Lefter, Walther Bild

**Affiliations:** 1 Department of Pharmacodynamics and Clinical Pharmacy, “Grigore T. Popa” University of Medicine and Pharmacy, 16 Universitatii Street, 700115 Iasi, Romania; dana.ababei@gmail.com (D.-C.A.); alexandru.vasincu@umfiasi.ro (A.V.); razvan.nicolae.rusu@gmail.com (R.-N.R.); 2 Center of Biomedical Research, Romanian Academy, Iasi Branch, 8 Carol I Avenue, 700506 Iasi, Romania; radu_lefter@yahoo.com (R.-M.L.); waltherbild@gmail.com (W.B.); 3 Department of Anaesthesiology and Intensive Therapy, “Grigore T. Popa” University of Medicine and Pharmacy, 16 Universitatii Street, 700115 Iasi, Romania; miblaj@yahoo.com; 4 Center for Advanced Research and Development in Experimental Medicine (CEMEX), “Grigore T. Popa” University of Medicine and Pharmacy, 16 Universitatii Street, 700115 Iasi, Romania; gabriela-dumitrita.s@umfiasi.ro; 5 Department of Physiology, “Grigore T. Popa” University of Medicine and Pharmacy, 16 Universitatii Street, 700115 Iasi, Romania

**Keywords:** renin–angiotensin, angiotensin II, Alzheimer dementia, ACE inhibitors, ARBs, aliskiren

## Abstract

The Renin–Angiotensin System (RAS) has attracted considerable interest beyond its traditional cardiovascular role due to emerging data indicating its potential involvement in neurodegenerative diseases, including Alzheimer’s dementia (AD). This review investigates the therapeutic implications of RAS modulators, specifically focusing on angiotensin-converting enzyme inhibitors (ACEIs), angiotensin receptor blockers (ARBs), and renin inhibitors in AD. ACEIs, commonly used for hypertension, show promise in AD by reducing angiotensin (Ang) II levels. This reduction is significant as Ang II contributes to neuroinflammation, oxidative stress, and β-amyloid (Aβ) accumulation, all implicated in AD pathogenesis. ARBs, known for vasodilation, exhibit neuroprotection by blocking Ang II receptors, improving cerebral blood flow and cognitive decline in AD models. Renin inhibitors offer a novel approach by targeting the initial RAS step, displaying anti-inflammatory and antioxidant effects that mitigate AD degeneration. Preclinical studies demonstrate RAS regulation’s favorable impact on neuroinflammation, neuronal damage, cognitive function, and Aβ metabolism. Clinical trials on RAS modulators in AD are limited, but with promising results, ARBs being more effective that ACEIs in reducing cognitive decline. The varied roles of ACEIs, ARBs, and renin inhibitors in RAS modulation present a promising avenue for AD therapeutic intervention, requiring further research to potentially transform AD treatment strategies.

## 1. The Role of the Renin–Angiotensin System in Alzheimer’s Dementia (Neuroprotection and Neurodegeneration)

The most common cause of dementia worldwide is AD, a neurodegenerative disease characterized by physiological changes caused by molecular changes. Clinically, it is accompanied by a loss of motivation, behavioral problems, poor care, disorientation, and deterioration of cognitive functions (memory, learning), with a gradual decline in intellectuality [1,2,3,4].

This form of dementia can be caused by different pathologies. The abnormal accumulation of beta-amyloid (Aβ) is considered the promoter of this disease, subsequently inducing tau protein hyperphosphorylation, neuroinflammation, oxidative stress, and synaptic transmission deficits; cholinergic neurotransmission occupies the primary place in this pathology. All these elements cause mitochondrial dysfunction, with neuronal damage leading to neuroinflammation and neurodegeneration, with an undeniable role in AD [5,6,7]. An important risk factor for dementia and neurodegenerative diseases is hypertension, in whose pathophysiology an important role is played by the upregulation of the RAS. Numerous studies support that the effective lowering of blood pressure through therapy or lifestyle changes contributes to the improvement of cognitive function, thereby reducing the risk of AD [8].

**Figure 1:**
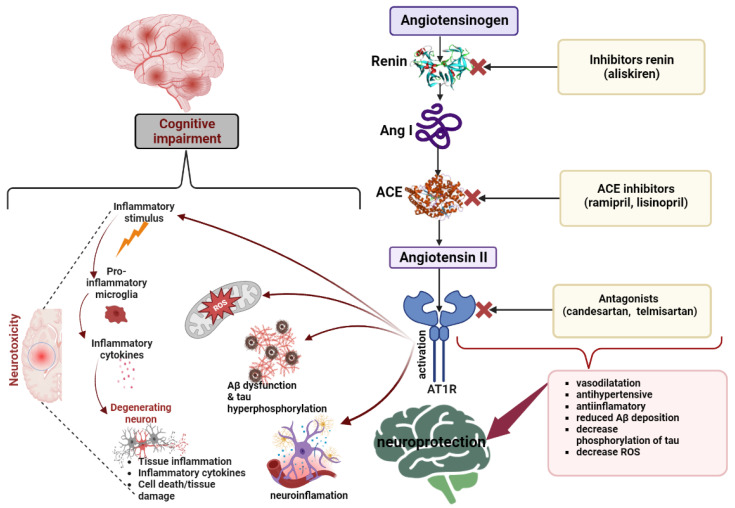
The angiotensinogen in the presence of renin is converted to angiotensin (Ang) I. Subsequently, Ang I in the presence of an angiotensin-converting enzyme (ACE) is converted to Ang II, and this activates AT1 receptor (AT1R) and AT2R. The activation of AT1Rs by Ang II causes a series of negative effects, some with repercussions on cognitive status, including the onset of dementia caused by neurotoxicity, neuroinflammation, oxidative stress, beta-amyloid deposits, and hyperphosphorylation of tau proteins. Compounds such as aliskiren inhibit renin, thus decreasing the synthesis of Ang I. Angiotensin-converting enzyme inhibitors (ACEIs) (like ramipril) reduce the potential for the transformation of Ang I into Ang II. AT1R blockers of Ang II (like candesartan) antagonize its effects at the receptor level. These modulators, through their actions, produce a series of therapeutical effects such as anti-inflammatory effects, vasodilatation, lowering blood pressure, reducing oxidative stress, and reducing amyloid deposits, thus providing neuroprotection.

Physiologically, brain Ang II is involved in numerous effects mediated by AT1 receptor (AT1R) and AT2R that are G protein-coupled receptors (GPCRs). The downstream activation of AT1R by Ang II is mediated by the involvement of mitogen-activated protein kinases (MAPKs), non-receptor tyrosine kinases (NRTK), phospholipases C, D and A2, and nicotinamide adenine dinucleotide phosphate (NADPH) oxidases (NOX), which induce a range of effects, such as vasoconstriction, hydrosaline retention, stimulation and increase in aldosterone synthesis, vasopressin release, induction of pro-inflammatory pathways, fibrosis and hypertrophy, synthesis of reactive oxygen species (ROS), and apoptosis. However, by activating AT2R, anti-inflammatory pathways, vasodilation, and promotion of cell survival are facilitated [9,10,11]. Figure 1 illustrates important elements of RAS key constituents that interact in a cascade-like manner to exert their effects not only on the cardiovascular system but also extending beyond it.

The complex Ang II/AT2R triggers the expansion of nerve cell projections known as neurites. This phenomenon, referred to as neurite outgrowth, indicates the hormone’s potential involvement in enhancing the growth and interconnectivity of nerve cells. This mechanism holds implications for diverse physiological and pathological processes within the brain. Nevertheless, these receptors encompass a wider range of functions, including the initiation of several signaling cascades that contribute to the upregulation of MAPKs, the promotion of neuronal differentiation, the facilitation of axonal regeneration, the induction of vasodilation, and the exertion of antifibrotic effects [11,12,13].

The inflammatory responses and apoptosis of neuronal cells caused by AT1R activation by Ang II may be due to the triggering of events involving MAPK p44/42 and C-Jun N-terminal kinase (JNK) [6,14]. The activation of angiotensin II type 1 receptor (AT1R) and subsequent events involving MAPK p44/42 (also known as ERK1/2) and JNK (c-Jun N-terminal kinase) signaling pathways can have significant implications for various clinical diseases, including those with potential links to Alzheimer’s disease (AD), such as hypertension and cardiovascular diseases, diabetes mellitus and diabetic complications, neuroinflammatory conditions or stroke, and ischemic injury [15].

The alteration of the brain RAS plays a key role in AD, as the local chronic activation of this system leads to the triggering of pathological processes of neuronal destruction, caused by an increased brain Ang II synthesis, producing impairment of cognitive function through nitric oxide (NO) synthesis involving AT2R subtypes [14,16]. Brain RAS stimulation was associated with an increased Ang II synthesis in rats, by showing low levels of antioxidant enzymes, while malondialdehyde (MDA) and superoxide dismutase (SOD) showed high values, proving that ROS generation causes neurodegeneration [14,17]. The effects of AT1R stimulation by Ang II associated with astrocyte or microglia activation would be due to the activation of MAPKs, phenomena which are observed in neuronal culture studies [9]. In AD, AT2R exerts opposite effects to AT1R, by reducing amyloid deposition and neuronal injury, effects which are mediated by an increased cerebral blood flow (CBF) [6].

The intracerebral administration of Aβ in the hippocampus of mice induced oxidative stress caused by the demineralization and oligomerization of AT2R under the action of transglutaminase. Tau phosphorylation and neurodegeneration are enhanced by the deficient action of AT2R-specific oligomers and dimers as they sequester the Gq11 protein, whose role is to inhibit cholinergic neurotransmission by blocking M1-type muscarinic receptors [18,19].

There is ample evidence that Ang II inhibits the release of acetylcholine (Ach), a neuromediator involved in AD pathology, while the pharmacological inhibition of Ach has been shown to antagonize the detrimental effects of scopolamine on cognitive function. On the other hand, the metabolism of Ang II leads to the obtainment of Ang IV, a metabolite that facilitates memory and its long-term potentiation, via insulin-regulated aminopeptidase (IRAP) and used as a neuroprotectant, thus contributing to improved cognitive function [6,14,20].

In preclinical studies, Ang II formation has been observed to interact with memory acquisition and in most clinical trials in which ACEIs have been administered, effects such as cognitive facilitation have been observed [21].

A number of memory-enhancing effects have been attributed to Ang IV resulting from Ang II conversion, a peptide that facilitates cognitive functions by acting as an agonist to the AT4 receptor subtype. Memory enhancement by Ang IV is promoted by glucose uptake at the neuronal level by modulating GLUT4 translocation at the cell surface [22]. Ang IV can bind to AT2R with beneficial effects on cognitive activity, as its administration in an animal model of AD for one month favored spatial memory improvement [23,24].

It has been shown that an increase in angiotensin-converting enzyme (ACE) activity occurs in AD, alongside changes in other components of the brain RAS. Numerous preclinical and clinical studies have shown that antagonists or Ang II AT1R blockers (ARBs) and ACE have beneficial effects by decreasing Ang II activity and increasing brain substance P, the latter being a substrate of ACE which in turn is involved in increasing the activity of neprilysin, an enzyme with a role in amyloid degradation and the reduction of brain inflammatory processes [10,20,25,26].

Under physiological conditions, the blood–brain barrier (BBB) is impermeable to Ang peptides, but in various pathological states this membrane becomes permeable, and Ang II may influence the facilitation of cerebral endothelial crossing, whose actions are mediated by AT1Rs [11,27].

Also, in AD, an increase in pro-inflammatory cytokines (interleukin (IL)-1β) induced by Ang II has been observed, whose actions affect the long-term potentiation (LTP) in the hippocampus of synaptic neurotransmitters involved in memory and learning, while the activation of AT1Rs by Ang II results in impaired memory. The correlation between Aβ and RAS is based on the fact that Ang II acts on γ-secretase, an enzyme that cleaves amyloid precursor proteins (APPs), thus suggesting that Ang II through AT1R activation is involved in AD pathology [11,12]. This major component of RAS also contributes to AD pathology through apoptosis phenomena, a major pathway of neuronal destruction, with ROS-generated apoptosis being induced by Ang II [28]. Other studies support the role of ACE in AD by degrading the Aβ1-42 peptide, in vivo but also in vitro, thus preventing plaque formation. This enzyme has also been associated with an improved immune response, a phenomenon observed in a transgenic mouse study, when cognitive decline was prevented due to the overexpression of this enzyme in myelomonocytes, amplifying resistance against Aβ forms [29].

Another key component of RAS is Ang-(1-7), for which a protective role against vascular ageing has been highlighted, as the endogenous synthesis of this peptide is reduced with ageing. Although it can be synthesized from Ang II under the action of its converting enzyme (ACE2), this heptapeptide has antagonistic actions towards Ang II, through its binding to Mas GPCRs [30]. The anti-inflammatory, vasodilator, antioxidant, antifibrotic, and anti-proliferative actions of Ang-(1-7) contribute to the protective effect against ageing [31]. The hippocampus, cortex, and basal ganglia are brain structures with a key role in cognitive function. This function could be attenuated by the activation of Mas receptors, which exhibit anti-inflammatory and antioxidant effects. For the primary ligand represented by Ang-(1-7) of Mas receptors to exert an effect, the presence of AT2R is required, and also Mas receptors are necessary for the beneficial effects of Ang-(1-7) [32].

## 2. The Effects of ACE Inhibitors in AD Pathology

Are ACEIs effective in patients with AD? What is the link between hypertension and AD? There is a lot of preclinical and clinical evidence supporting that hypertension can cause cognitive impairment. The disruption of BBB and some neurovascular structures leads to increased oxidative stress and neuroinflammation, phenomena that facilitate cognitive impairment [11]. The likelihood of developing dementia in the presence of hypertension and stroke is reinforced by the fact that these diseases have profound effects on cerebral circulation [11,33].

It appears that some ACEIs, and in particular captopril and perindopril, contribute to decreased Ang II synthesis, not only peripherally but also in the brain. This phenomenon has also been observed in clinical trials, where male patients treated for 24 weeks with captopril achieved not only lower blood pressure but also improved mental activity, but not the same effect was observed with propranolol [21].

In numerous studies, it has been observed that the inhibition of brain RAS by the administration of ACEIs can reduce the cognitive decline present in AD or other neurodegenerative diseases [26,34]. Other studies argue that, in fact, the use of ACEIs may actually increase the incidence of AD [25].

Based on preclinical studies in which ACEIs prevented neuronal damage in animal models of AD, some researchers have tested this hypothesis in the Japanese population with AD and hypertensive patients at the same time. When using ACEIs that crossed BBB, the beneficial role of the existing correlation between brain RAS and neurodegenerative disorders in memory and learning processes, with improved cognitive functions, probably through the inhibition of Ang II formation, as well as cholinergic neurotransmission, has been observed [20,26,34,35].

The effectiveness of these inhibitors depends very much on the degree of lipophilicity, which will allow BBB crossing or not. For example, some studies claim that captopril would have very poor lipid solubility, while other studies show that its administration in mice and human subjects, respectively, can penetrate into the cerebrospinal fluid (CSF). Lisinopril, perindopril, and enalapril are other inhibitors with central nervous system (CNS) affinity, inhibiting at this level the effects of brain RAS, with enalapril and perindopril demonstrating antioxidant activity in animal models of AD, by attenuating ischemic brain edema and preventing cognitive impairment caused by oxidative stress [6,36,37].

Lisinopril has positive effects in learning and memory enhancement, cerebral cholinergic activity, attenuation of inflammation, and reduction of oxidative stress in a murine model of intracerebroventricular streptozotocin-induced dementia of AD. Its favorable impact was associated with the modulation of peroxisome proliferator-activated receptor-γ (PPAR-γ) [38]. In a study by Thomas et al., lisinopril demonstrated a notable enhancement in addressing learning and memory deficits as well as climbing impairment in a *Drosophila melanogaster* model of AD. The observed beneficial influence of lisinopril on physical performance could be attributed, at least partially, to a marked decrease in ROS levels within the thoraces of AD flies treated with lisinopril [39].

The efficacy of ACEIs on memory deficits differs between representatives, with perindopril improving cognitive performance in preclinical studies in a mouse model of AD, while enalapril and imidapril showed no significant effects on behavior in a dementia model [40]. Table 1 summarizes the protective mechanisms through which ACEIs act in the prevention of AD.

Increased ACE activity has been observed in AD patients, and the decrease in the levels of this enzyme can be modulated by the administration of ACEIs that cross the BBB, thereby slowing the rate of decline in cognitive function [26]. In vitro evidence has shown that some ACEIs such as captopril and perindopril have important roles in preventing neuroinflammation and neurodegeneration due to decreased NO synthesis, with perindopril being able to interfere with astrocytes and microglia for this effect. Although enalapril in in vitro studies inhibited the formation of zinc-bound Aβ(1-16), which could induce AD due to its neurotoxicity, in in vivo studies, the efficacy of enalapril has been questioned due to its inability to cross the BBB [6]. The efficacy of ACEIs has been demonstrated in several in vivo studies in the preclinical area in animal models with memory impairment induced by various agents. Based on the fact that cholinergic neurotransmission plays an important role in cognition, numerous preclinical studies demonstrated that scopolamine-induced amnesia could be prevented by the administration of ACEIs, which, due to the inhibition of the angiotensin converting enzyme, suppressed MDA levels, thus attenuating oxidative stress at brain level [26]. Also, our group of researchers demonstrated that ramipril and captopril administration prevented the deterioration of cognitive functions in mice on a model of dementia induced by an anticholinergic agent (scopolamine) [41], whereas the cortical cholinergic deficit was not restored by enalapril administration, due to its low ability to cross the BBB [6,42].

There are a number of arguments supporting the effects of ACEIs in cognitive facilitation. The administration of an ACEI could favor the potentiation of Aβ degradation, since ACE degrades neprilysin, an enzyme involved in its degradation, whose increased activity is facilitated by substance P [21]. Even though ACEIs are currently used for conventional therapy of hypertension, studies in animal models have shown that, in the case of those compounds that can cross the BBB (perindopril for example), cognitive function has been rescued in AD mice [11,40].

However, in clinical trials, the central effects of ACEIs have been shown to be limited, with cognitive performance improving only in the first 9 months of therapy but not thereafter [43].

In middle age, it has been found that hypertension can be associated with mild cognitive impairment to dementia, whose risk of developing AD can be slowed by the administration of ACEIs due to the inhibition of Ang II formation with lower blood pressure values, and at brain level it can exert neuroprotective effects if it crosses the BBB [44].

The link between hypertension and susceptibility to neurodegeneration has been studied and analyzed in various preclinical studies. ACE inhibition of Ang I to Ang II by ACEIs promoted neuroprotection. The association between RAS and brain deterioration (dementia) has been confirmed by the actions of ACEIs through the positive modulation of cognitive functions, with memory being improved. In an experimental model of lipopolysaccharide (LPS)-induced neuroinflammation in hypertensive rats, neurodegeneration was observed. The involvement of Ang in LPS-induced neuroinflammation was confirmed by oral administration of perindopril for 15 days at a dose of 0.1 mg/kg b.w., the efficacy of which was observed in the significant improvement in cognitive functions, particularly memory (Table 2) [34].

According to a meta-analysis, ACEIs have been reported to play an important role in AD by improving cerebral blood flow, increasing Ach concentration, and reducing pro-inflammatory activity and oxidative stress [45].

**Table 1:** Protective mechanisms of ACEIs in the prevention of AD.

Mechanism of AD Pathogenesis	Mechanism of Protection	Effect	Reference
Cognitive decline	Inhibition of brain RAS	Decrease in ACE activity Reduction of cognitive decline	[26,34]
Cholinergic hypothesis	Inhibition of Ang II formation Suppression of MDA levels → alleviation of oxidative stress at brain level	Improvement of cognitive functions → Prevention of scopolamine-induced amnesia	[20,26,34,35]
Formation of Aβ peptide aggregates in the brain [46]	Increase in neprilysin activity	Favoring the potentiation of Aβ degradation	[21]

Aβ: β-amyloid; ACE: angiotensin-converting enzyme; Ang II: Angiotensin II; MDA: malondialdehyde; RAS: renin–angiotensin system; →: direct involvement.

**Table 2:** The effects on biological pathways for potential therapeutic insights of ACEIs in AD.

Compound	Effect on Biological Pathway	Disease Model and Species	Reference
Captopril Perindopril	Prevention of neuroinflammation and neurodegeneration due to decreased NO synthesis	In vitro studies	[6]
Perindopril	Interference with astrocytes and microglia	In vitro studies	[6]
Enalapril	Inhibition of the formation of neurotoxic zinc-bound Aβ(1-16)	In vitro studies	[6]
Enalapril Perindopril	Antioxidant activity → Attenuation of ischemic brain edema Prevention of cognitive impairment caused by oxidative stress	In vivo studies	[6,36,37]
Enalapril	Cortical cholinergic deficit was not restored due to its low ability to cross the BBB	In vivo studies	[6,42]
Perindopril	Improvement of cognitive performance	In vivo study	[40]
Perindopril	Significant improvement in cognitive functions	In vivo model of LPS-induced neuroinflammation	[34]
Ramipril Captopril	Prevention of deterioration of cognitive functions	In vivo model of dementia induced by scopolamine	[41]
Lisinopril	Positive effects in learning and memory enhancement, cerebral cholinergic activity, attenuation of inflammation, and reduction of oxidative stress ← modulation of PPAR-γ	In vivo model of murine model of intracerebroventricular streptozotocin-induced dementia of AD	[38]
	Enhancement in addressing learning and memory deficits as well as climbing impairment ← marked decrease in ROS levels within the thoraces of AD flies	In vivo study—Drosophila melanogaster model of AD	[39]
Captopril	Decrease in Ang II synthesis, not only peripherally but also in the brain Improvement of mental activity	Clinical trials—male patients treated for 24 weeks	[21]
ACEI	Improvement of cognitive performance only in the first 9 months of therapy but not thereafter	Clinical trials	[43]

Aβ(1-16): β-amyloid(1-16); Ang II: Angiotensin II; BBB: blood–brain barrier; LPS: lipopolysaccharide; NO: nitric oxide; PPAR-γ: peroxisome proliferator-activated receptor-γ; →: direct involvement; ←: causality relation.

## 3. The Effects of Ang II AT1R Antagonists in AD Pathology

Since the discovery that the ACE1/Ang II/AT1R axis is responsible for the injurious effects of the brain and systemic RAS, and is involved, besides other factors, in the development of AD [47], blocking this axis by various pharmacological agents has become a topic of interest for researchers. Hence, an important class of drugs that may possess the potential to diminish injurious effects on memory and cognition in AD refers to Ang II AT1R antagonists (ARBs), especially since studies have shown that elevated levels of both Ang II and AT1Rs were registered in the brains of AD patients [6,48].

ARBs are medicines used in the treatment of arterial hypertension. There are eight representatives of this class used in therapy: losartan, candesartan, eprosartan, irbesartan, olemsartan, telmisartan, valsartan, and azilsartan. The first commercialized ARB was losartan in the late 1990s [49], and the one most recently introduced on the market was azilsartan, approved by the Food and Drug Administration (FDA) in 2011 [50]. Within the class, although they have certain parts in common, the chemical structures of the representatives are different, which gives them different pharmacokinetic profiles and different affinity for AT1Rs [50,51]. ARBs manifest their action by antagonizing the effects of Ang II via the AT1R pathway. The latter are widespread in the brain and, being an important potentiator of the NADPH-oxidase complex, their activation results in pro-oxidative and pro-inflammatory effects; superoxide formation, cerebral blood flow regulation, cognitive impairment, and vasoconstriction have also been associated with AT1R activation [52]. Multiple studies have demonstrated the positive cognitive effects of ARBs in AD or other pathologies, with these drugs showing vascular protective effects and neuroprotective effects. It seems that in terms of cognitive benefits, ARBs act in two ways: on the one hand, directly by blocking AT1Rs and, implicitly, the harmful pathway, and, on the other hand, indirectly by allowing the stimulation by free Ang II of AT2Rs [50], whose activation is thought to counterbalance the AT1R-mediated actions [52].

### 3.1. Telmisartan

Telmisartan showed neuroprotective effects in some in vitro models of neurotoxicity induced by IL-1β [53] and glutamate exposure [54]. In both studies, telmisartan was found to be the most potent compared to the other ARBs studied, and in terms of PPAR-γ activation, it was present in cultured rat primary cerebellar granule cells exposed to glutamate [54] but was absent from human SK-N-SH neuroblasts exposed to IL-1β [53]. Torika et al. [55] showed that telmisartan can reduce the expression of inflammatory markers (NO, tumor necrosis factor-α (TNF-α), IL-1β) in BV2 cells stimulated with LPS, effects that were not observed in unstimulated BV2 cells. Anti-inflammatory effects with reduced expression of IL-1β, TNF-α, nuclear factor (NF)-κB activity, as well as anti-inflammatory IL-10, were observed in BV2 cells stimulated by a soluble Aβ oligomer [56]. Elkahloun et al. [57] found that telmisartan possesses neuroprotective effects also through other mechanisms besides AT1R blockade or PPAR-γ activation. In BV2 cells lacking AT1R expression or PPAR-γ activation, telmisartan was able to prevent the increase in LPS-induced inflammatory markers, and reduce the expression of numerous genes associated with inflammation, apoptosis, and neurodegenerative disorders, while downregulating the expression of genes linked to oncological, neurodegenerative, and viral diseases [57].

Telmisartan is among the most studied ARBs in vivo on numerous animal models of neurovascular and cognitive impairment. The intranasal administration of telmisartan both short-term (1–2 months) and long-term (5 months) reduced cortical and hippocampal amyloid burden as well as glial activation in 5XFAD mice [55,58]. The Aβ deposition was also reduced by telmisartan in ddY mice intracerebroventricular-injected with Aβ1–40, improving the cognitive decline induced by Aβ [59,60], and reducing the expression of TNF-α and inducible nitric oxide synthase (iNOS) [60]. In addition to the well-known AT1R blocking mechanism, the potential of telmisartan to manifest its neuroprotective and anti-inflammatory effects also through PPAR-γ activation has been studied and demonstrated in numerous studies on various preclinical in vivo models [59,60,61,62,63,64,65]. Because aluminum is considered one of the factors involved in AD pathogenesis, Khalifa et al. [66] evaluated the effects of telmisartan on the cognitive impairment in rats treated with aluminum chloride. The oral administration of telmisartan showed beneficial effects on learning and memory decline produced by aluminum and resulted in reductions in MDA, TNF-α, NF-κβ, Aβ1-42, and phosphorylated tau protein levels, all of which were elevated by aluminum treatment [66]. Telmisartan’s potential to reduce cognitive impairment associated with AD is also suggested by its ability to attenuate the increased acetylcholinesterase hippocampal levels produced by aluminum administration [66]. Furthermore, telmisartan exhibited neuroprotective effects, preventing and attenuating cognitive impairment associated with obesity [67], diabetes mellitus (DM) [63], and acute and chronic stress [68] in different preclinical rodent models. A pharmacokinetic brain PET study in rhesus macaques showed that after bolus administration of telmisartan (1 mg/kg b.w.), it penetrated the brain in a low proportion, but it was sufficient to block AT1Rs and was evenly distributed in the entire brain [69].

As the pathogenic mechanisms and features of AD cannot be completely reproduced in animal models [6], the results of clinical trials evaluating the effects of telmisartan on cognitive impairment are not entirely conclusive. Hence, an analysis of two large randomized controlled trials (the ONTARGET and TRANSCEND trials), which had as secondary outcomes the assessment of cognitive decline after treatment with telmisartan, ramipril, a combination of the two, or placebo [70,71], showed a non-significant difference in cognitive impairment occurring in studied groups of high-vascular-risk patients [72]. Kume et al. [73] compared the effects of telmisartan vs. amlodipine on cognition and cerebral blood flow in 20 patients with hypertension and probable AD. While amlodipine led to worse cognitive performance after 6 months of treatment, telmisartan showed no change or improved performance, as well as an improvement in regional cerebral blood flow in multiple regions of the brain [73]. The FDG-PET study of Imabayashi et al. on four hypertensive AD patients regarding brain glucose metabolism suggests that telmisartan may have the ability to inhibit the short-term decline of glucose metabolism in the anterior olfactory nucleus of the olfactory tract in AD patients’ brains [74]. Promising results were obtained in the Liu et al. [75] study, where hypertensive patients with type 2 diabetes mellitus (T2DM) treated with telmisartan (compared to patients treated with ARBs other than telmisartan) manifested a lower risk of dementia diagnosis or dementia diagnosis with ischemic stroke as a competing risk and with all-cause mortality as a competing risk. Also, a randomized clinical trial, with approximately 7 years of follow-up, identified a synergistic effect between telmisartan and rosuvastatin to reduce the risk of dementia occurrence and the cognitive impairment evolution, especially in hypertensive patients with the APOE ɛ4 allele compared to those without this allele [76]. Interestingly, telmisartan was associated with greater beneficial effects and a reduced incidence of AD in African Americans, but not in non-Hispanic White or European Americans [77,78]. Another clinical trial, that has not yet published its results, evaluates the effects of telmisartan in the prevention of AD, with the determination of the dose required for CNS penetration and AD-specific biomarkers (NCT02471833) [79]. The above-mentioned effects of telmisartan in AD are summarized in Table 3.

### 3.2. Candesartan

The effects of candesartan have been evaluated in several studies on in vitro models of AD. The results of the Choi et al. [80] study showed that candesartan, via the P13K pathway, was able to restore the proliferation of neural stem cells previously inhibited by the Aβ25-35 oligomer. In BV2 cells stimulated with LPS, candesartan treatment led to decreases in the expression of pro-inflammatory markers (iNOS, cyclooxigenase-2 (COX-2)) and the promoted expression of Arginase-1 (Arg-1), an anti-inflammatory marker, along with an enhanced Aβ1-42 uptake by microglia [81]. The same study also observed that candesartan decreased NO, TNF-α, and transforming growth factor-β1 (TGF-β1), but it did not affect the IL-1β levels [81].

Elkahloun et al. studied the effects of candesartan on primary neuronal cultures treated with glutamate in excitotoxic concentrations. The results showed the neuroprotective effects of candesartan, the agent preventing alterations in several transcripts that were up- or downregulated by glutamate [82]. Glutamate excess is associated with neuronal injury and is considered to be a factor in the pathogenesis of AD [6].

Numerous preclinical studies have evaluated the effects of candesartan in animal models of AD. Therefore, the intranasal administration of candesartan to 5XFAD mice resulted in a significant decrease in the expression levels of amyloid burden, but only in the hippocampus layer, not in the cortical layers of treated animals [81]. In a mouse model of memory impairment induced by the intracerebral administration of streptozotocin, candesartan improved spatial memory, decreased oxidative stress [83], and restored acetylcholinesterase activity [84]. Candesartan also efficiently prevented neuroinflammation induced by LPS, increased AT2R expression [85], and prevented neuroinflammation and astrocyte and microglial activation in the brain of a rat model of chronic hypertension [86]. The results of the Villapol et al. [87] study suggest that candesartan (and telmisartan), manifests its action both by blocking AT1Rs and by activating PPAR-γ. On the other hand, in the Trigiani et al. [88] study on APP mice, candesartan reduced neuroinflammation and restored the function of the endothelial and smooth muscle, but with no significant impact on impaired cognitive function and amyloid plaque load.

A randomized clinical trial on hypertensive patients over 65 years old with early cognitive impairment suggests that candesartan is associated with an improvement in executive function in studied patients, exceeding the benefits produced by lisinopril and hydrochlorothiazide. Reducing inflammation, restoring central endothelial function, and a lack of AT2R inhibition with prevention of neuronal degeneration seem to be related to candesartan’s superior effects in cognitive impairment [89,90]. Another, more recent, randomized clinical trial evaluated the safety and efficacy of candesartan treatment (1 year) in non-hypertensive adults with prodromal AD. Candesartan was found to be safe and to be associated with a lower brain amyloid deposition by increases of Aβ40 and Aβ42 in CSF but with no significant modifications in tau protein levels. Also, candesartan was found to enhance connectivity in subcortical brain networks and to have beneficial cognitive effects [91]. The above-mentioned effects of candesartan in AD are summarized in Table 4.

### 3.3. Losartan

Losartan’s impact on memory and cognition was explored in several in vivo studies, involving different animal models or humans. In their study proposing the intranasal administration of losartan to avoid hypotensive adverse effects that might occur in normotensive patients, Danielyan et al. [92] studied the effect of losartan in the APP/PS1 transgenic mouse model of AD. Two months of intranasal losartan treatment showed neuroprotective and anti-inflammatory effects by decreasing Aβ plaques, decreasing IL-12 p40/p70, IL-1β and granulocyte-macrophage colony-stimulating factor (GM-CSF), and increasing IL-10 [93]. A reduction of Aβ plaques was also observed in a rat model of chronic hypertension treated with intranasal losartan. The same study showed improvements in neurological deficits and neuroinflammation, increases in choroid plexus cell proliferation, neurogenesis, cell survival, and IL-10 levels in the brain, and decreased mortality [93]. Similar results were obtained with the intraperitoneal administration of losartan in APP/PS1 mice [94].

However, Papadopoulos et al. [95] found no changes in Aβ levels in A/T mice treated with 10 mg/kg b.w./day losartan, for three months. Also, losartan treatment did not restore cognitive deficits and did not attenuate astroglia activation, although it did improve cerebrovascular reactivity in A/T mice [95]. Onigali et al. [96] evaluated the therapeutic potential of losartan to both cure (3-month treatment) and prevent (10-month treatment) signs of AD in adult and aged APP transgenic mice. Losartan effectively prevented the onset of cognitive dysfunction (learning and memory deficits) and dilatory deficits in adult APP mice treated preventively. This protection was attenuated in aged APP mice, treated for 3 months with losartan, but the agent improved memory performance even though no significant benefits on learning deficits were observed. These positive effects were not accompanied by decreases in soluble Aβ species or plaque load. In both adult and old APP mice, losartan significantly decreased the cerebrovascular and cortical AT1R elevated levels [96].

Regarding clinical research, a recent randomized placebo-controlled trial (the RADAR trial) studied the effects of losartan in clinically diagnosed mild-to-moderate AD patients (aged 55 years or older) [97]. After 12 months of treatment with losartan, they found no decrease in the rate of brain atrophy in the studied patients. The authors considered the possibility that losartan may not have crossed the BBB to the extent expected [97]. The above-mentioned effects of losartan in AD are summarized in Table 5.

### 3.4. Olmesartan

Olmesartan was found to exhibit protective effects in Aβ-induced cellular senescence and neurotoxicity on M17 neuronal cells, by reducing ROS and MDA elevated levels, as well as the expression of some senescence biomarkers [98].

In high-salt and high-cholesterol diet-fed mice, olmesartan attenuated cognitive decline and improved cognitive function, increased the messenger ribonucleic acid (mRNA) expression of a neuroprotective factor, methyl methanesulfonate sensitive 2, and decreased superoxide anion production in the brain [99]. Also, olmesartan treatment led to a reduction of oxidative stress in microvessels and to the attenuation of the cerebrovascular and cognitive impairment in APP23 mice, an AD transgenic mice model, without decreases in the Aβ brain level [100]. In intracerebrovascular Aβ1-40-injected mice, olmesartan was able to prevent vascular dysregulation and improve cognitive function [100]. In Dahl salt-sensitive rats fed with a high-salt diet, olmesartan attenuated cognitive decline, significantly decreased the BBB microvessels’ permeability, and reduced hippocampal levels of Ang II, all of which were elevated by the high-salt diet [101]. Prevention of BBB disruption, as well as a reduction of oxidative stress with significant improvements in cognitive impairment, were also observed in 5XFAD mice with transient cerebral ischemia caused by bilateral common carotid artery occlusion treated with olmesartan [102]. The effects of olmesartan in AD are summarized in Table 5.

### 3.5. Valsartan

In vitro, valsartan was able to reduce the oligomerization of Aβ peptides into high-molecular-weight oligomeric peptides, which is correlated to cognitive impairment [103], and it was found to increase dendritic spine density in developing and mature hippocampal neurons, which is associated with learning and memory [104].

Similarly, in the case of this ARB representative, non-human in vivo studies support the beneficial effects of the class. Thus, in Tg2576 mice, valsartan attenuated spatial memory impairment related to Aβ accumulation [103]. Also, in another model of AD-type dementia (rats treated with aluminum trichloride and D-galactose), cognitive benefits were supported by valsartan’s ability to alleviate oxidative stress and restore cholinergic function [105]. A reduction of oxidative/nitrosative stress and the potentiation of the brain’s defensive antioxidant system produced by valsartan have also been observed in rats intracerebroventricular-injected with streptozotocin [106]. A recent study assessed the effects of sacubitril/valsartan (a relatively new antihypertensive combination) in comparison with valsartan alone on a rat model of AD (aluminum-induced). The results showed that valsartan alone improved AD symptoms and did not increase the risk of developing AD, while the sacubitril/valsartan combination worsened the condition of the treated animals and increased their risk of developing AD [107].

Regarding clinical research, improvements in episodic memory have been observed, without influence on other tests of cognitive function, in elderly hypertensive patients treated with valsartan [108,109]. These effects are summarized in Table 6.

### 3.6. Irbesartan

In LPS-stimulated human brain microvascular endothelial cells, irbesartan reduced the endothelial permeability and restored the occluding expression, both impaired by LPS, and it seems that these effects were mediated via the NF-κB/Myosin light chain kinase (MLCK)/Myosin light chain (MLC) signaling pathway [110]. Likewise, Schupp et al. showed that irbesartan can stimulate the PPAR-γ activity [111].

In vivo, irbesartan was also found to reduce the BBB permeability, restore the occluding expression, reduce the expression of some inflammatory mediators, and improve the depressive-like behavior in an LPS-treated mice model [110]. Antidepressant effects as well as an increase in brain 5-hydroxytryptamine (5-HT) levels and a decrease in oxidative stress have been attributed to irbesartan treatment also in a mouse model undergoing various chronic mild stress procedures [112]. In AD-like pathology induced by aluminum in rats, irbesartan was found to mitigate cognitive impairment and to significantly reverse the amyloidogenesis [113] (Table 6).

### 3.7. Azilsartan

Azilsartan medoxomil, the latest ARB approved for hypertension treatment [114], was found to prevent and reverse cerebrovascular remodeling and dysfunction, as well as to decrease blood glucose levels in diabetic GK rats [115]. Iwanami et al. [116] suggest that the AT2R stimulation may be involved in the preventive effect of azilsartan on cognitive impairment in a mouse model of vascular dementia (induction of bilateral common carotid artery stenosis) since these beneficial effects of azilsartan were weaker in the same model of AT2 KO mice. In the aluminum chloride-induced neurotoxicity model of rats, azilsartan (but also perindopril or a perindopril/azilsartan combination) led to improvements in cognitive decline, as well as decreases in TNF-α, MDA, acetylcholinesterase, and Aβ-42 levels [117]. The effects of azilsartan in AD are summarized in Table 6.

### 3.8. Eprosartan

The results of the OSCAR study, a 6-month observational study on hypertensive patients over 50 years old, showed that eprosartan treatment was correlated with a modest overall improvement in the Mini-Mental State Examination score. This improvement was correlated with blood pressure reduction, and the biggest improvements were observed in older patients initially characterized by lower scores [118]. These effects were maintained after a 12-month follow-up of 3600 patients from the previous study under eprosartan-based therapy [119] (Table 6).

**Table 6:** The effects of other ARBs in various pathological aspects of AD.

Compound	Effect on Biological Pathway	Disease Model and Species	Reference
Valsartan	Reduction of the oligomerization of Aβ peptides into high-molecular-weight oligomeric peptides	In vitro study	[103]
	Increase in dendritic spine density	In vitro study—developing and mature hippocampal neurons	[104]
	Attenuation of spatial memory impairment related to Aβ accumulation	In vivo study—Tg2576 mice	[103]
	Alleviation of oxidative stress Restoration of cholinergic function	In vivo study—rats treated with aluminum trichloride and D-galactose	[105]
	Reduction of oxidative/nitrosative stress Potentiation of the brain’s defensive antioxidant system	In vivo study—rats intracerebroventricular-injected with streptozotocin	[106]
	Improvement in episodic memory No influence on other tests of cognitive function	Clinical trial—elderly hypertensive patients	[108,109]
Irbesartan	Reduction of the endothelial permeability Restoration of the occluding expression	In vitro study— LPS-stimulated human brain microvascular endothelial cells	[110]
	Reduction of the BBB permeability Restoration of the occluding expression Reduction of the expression of some inflammatory mediators Improvement of the depressive-like behavior	In vivo study—LPS-treated mice model	[110]
	Increase in brain 5-HT levels Decrease in oxidative stress	In vivo study—mouse model undergoing various chronic mild stress procedures	[112]
	Mitigation of cognitive impairment Reversal of the amyloidogenesis	In vivo study—AD-like pathology induced by aluminum in rats	[113]
Azilsartan	Prevention and reversal of cerebrovascular remodeling and dysfunction Decrease in blood glucose level	In vivo study—diabetic GK rats	[115]
	AT2R stimulation → Prevention of cognitive impairment	In vivo study—mouse model of vascular dementia	[116]
	Improvement in cognitive decline Decrease in TNF-α, MDA, acetylcholinesterase, and Aβ-42 levels	In vivo study—aluminum chloride-induced neurotoxicity model of rats	[117]
Eprosartan	Modest overall improvement in the Mini-Mental State Examination score	Clinical trial OSCAR—hypertensive patients over 50 years old	[118]

Aβ: β-amyloid; AD: Alzheimer’s dementia; AT2R: AT2 receptor; BBB: blood–brain barrier; 5-HT: 5-hydroxytryptamine; LPS: lipopolysaccharide; MDA: malondialdehyde; TNF-α: tumor necrosis factor-α; →: direct involvement.

At the medication class level, retrospective or prospective cohort studies have evaluated the risk of developing AD or dementia in patients treated with long-term ARBs. Thus, Barthold et al. [120] found that ARBs used concomitantly with statins (pravastatin or rosuvastatin) can reduce the risk of AD and related dementias to a greater extent compared to statins combined with other antihypertensives, which do not influence the RAS, in older Americans. Among Hispanics, this reduction in incidence was not observed [120]. Also, a recent retrospective cohort study in a Korean population showed that BBB-crossing ARBs (such as azilsartan, candesartan, telmisartan, or valsartan) used for a long time were correlated with a significantly lower risk of AD development with an additive duration–response relationship [121]. Compared with other antihypertensive or cardiovascular disease drugs, ARBs were associated with significant reductions in the incidence and progression of AD and dementia in a predominantly male population over 65 years diagnosed with cardiovascular disease [122]. During a follow-up period of 12 years, Kuan et al. [123] found that all-cause dementia risk was almost 40% lower in patients taking ARBs than in patients not treated with ARBs; also, both ACEIs and ARBs prevented vascular dementia development, but not AD, in T2DM patients with hypertension. On the other side, in hypertensive Asian patients, long-term use of ARBs was not significantly correlated with a decrease in AD risk [124].

Studies comparing the effects of ARBs and ACEIs in elderly patients with cognitive impairment or AD found that ARBs outperformed ACEIs towards brain structure and performance in memory and cognition tests [125]. In patients with T2DM, ARBs seem to be associated with a slower rate of brain atrophy, compared to ACEIs [126]. Also, in AD non-carriers of APOE ε4 patients, ARB users showed greater memory preservation as well as a greater attention/psychomotor processing speed, especially compared to non-BBB-crossing ACEI users [127]. Ho et al. [128] also found an association between BBB-crossing ARBs and greater memory performance and a lower white matter hyperintensity volume over time, compared to other antihypertensive drugs in older adults without dementia. Nation et al. suggest that ARB users present a lower rate of incidence and progression of dementia, compared to other antihypertensive users or no antihypertensive treatment, showing mitigation in P-tau accumulation and cerebral amyloidosis, by attenuation of cerebrospinal fluid Aβ1–42 decreased levels [129]. On the other hand, while in older patients with normal cognition, ARBs were correlated with a slower rate of accumulation of Aβ in the cortex compared to ACEIs, none of the drug classes were associated with influencing Aβ accumulation rates in patients diagnosed with AD dementia or mild cognitive impairment [130].

The results of a meta-analysis suggest that, in hypertensive patients, the use of any antihypertensive medication can reduce the risk of dementia development, but found no significant differences between drug classes [131]. On the other hand, a more recent meta-analysis showed that, compared to ACEIs, ARB use can be associated with significant decreases in the risk of AD and any dementia development [19].

## 4. Neuroprotective Effects of Renin Inhibitors

Renin inhibitors are a class of drugs that target the RAS to lower blood pressure. Some key findings concerning those drugs:

- A review article from 2020 discusses the development of renin inhibitors from several decades ago to the present day, including both peptides and nonpeptides. The article notes that, despite extensive research, renin inhibitors are still struggling to find a niche in antihypertensive therapy [132].
- A Cochrane systematic review from 2020 compared the effectiveness of renin inhibitors to ACEIs for primary hypertension. The review found that renin inhibitors reduce blood pressure more than placebo, with the magnitude of this effect thought to be similar to that for ACEIs. However, the direct involvement of renin inhibitors in neurodegenerative diseases is not discussed [133].

Overall, while renin inhibitors have shown promise in lowering blood pressure and treating conditions like diabetic nephropathy, more research is needed to fully understand their clinical utility and effectiveness compared to other treatments like ACEIs.

Renin inhibitors are a type of medication that blocks the RAS, which is involved in regulating blood pressure and fluid balance in the body. There is some evidence to suggest that RAS inhibitors, such as ACEIs and ARBs, may have beneficial effects in AD. The mechanisms by which these drugs affect AD are not yet fully understood, but studies using *Drosophila* models of AD have shown that ACEIs and ARBs can suppress neuronal cell death and memory defects, suggesting that they may target the amyloid pathway [134,135].

ACEIs and ARBs have been found to have beneficial effects in AD by reducing the incidence and progression of dementia. The RAS is now known to underlie the successful treatment of almost 50% of patients in cardiovascular medicine, with possibilities of extension to DM, AD, and cancer [136]. A review article suggests that the pathogenesis of chronic kidney disease may cause or exacerbate AD, especially via the RAS [137], while another revealed the reno-protective potential of renin inhibition [138].

In vivo studies have also shown that ACEIs have protective effects against AD. The RAS is involved in the pathophysiology of AD, and an increased expression of ACE has been shown to aggravate AD [137]. Overactivation of brain RAS has been associated with the initiation and progression of AD through the increased production of amyloid beta and oxidative stress resulting in cognitive impairment [139].

Thus, direct renin inhibition seems to be a promising therapeutic strategy for subcortical vascular dementia [140].

Renin may have broader actions in the brain and may indicate a potential for interaction with the (pro)renin receptor or production of a ligand for non-AT1Rs/AT2Rs [141]. This suggests that renin inhibitors possess a unique and distinct mechanism of action compared with the other two therapeutic classes [142].

Several studies demonstrated the reno-protective potential of renin inhibition [138]. These data support a new paradigm for the genetic control of RAS activity in the brain by a coordinated regulation of the renin isoforms, with expression of renin-b tonically inhibiting the expression of renin-a under baseline conditions [143].

Furthermore, the aliskiren effects may involve downregulation of renin expression induced by Aβ [144]. These renin inhibitors are therefore potential therapeutic agents for the treatment of hypertension and related cardiovascular diseases, and their subsequent effects in the initiation and evolution of neurodegenerative diseases [145].

Overall, while there is some evidence to suggest that RAS inhibitors, such as ACEIs and ARBs, may have beneficial effects in AD, more research is needed to fully understand their mechanisms of action and potential therapeutic uses.

## 5. Perspectives in the Prevention and Treatment of AD (General and RAS-Related)

Currently, widely used RAS modulators, especially for cardiovascular pathology, can improve various body functions. Even if they cannot be indicated as a therapy in AD, they can still be targeted as an adjuvant medication through their indirect actions. Most clinical cases of AD are accompanied by co-morbidities requiring polymedication. ACEIs have demonstrated antioxidant activity, oxidative stress being the precursor of many pathologies, including AD, which shows that their long-term use may generate not only antihypertensive but also other effects [26].

Preclinical studies support that ACEIs could be useful as a therapeutic strategy to improve cognitive dysfunction in hypertensive or even normotensive patients due to the decrease in Ang II formation, which is involved in neuroinflammation [34].

Comparing the effect of ACEIs with that of ARBs, a recent meta-analysis revealed that ARBs may have a protective role against dementia in general, but also against AD, probably due to the blocking of AT1Rs only, while the AT2R signalling remains intact. This result should be considered in future studies involving antihypertensive medication and especially RAS modulators, paying particular attention to cognitive impairment [19]. The two therapeutic classes have different effects on amyloid processing, with studies reporting that there are differences in terms of neurodegeneration phenomena, with less atrophy observed with the use of antagonists [125,127].

Of the Ang II AT1R antagonists, candesartan demonstrated neuroprotective effects on rat neuronal cultures. The results of this study reported that this antagonist significantly prevented glutamate-induced upregulation, its excitotoxicity playing an important role in β-amyloid metabolism. Due to the fact that this compound prevents inflammation as well as changes in APP processing, this report argues for the inclusion of candesartan as a drug of choice in early dementia therapy [82].

Currently, there are a number of approved antihypertensive drugs such as perindopril, candesartan, and telmisartan in phase II clinical trials with the aim of repurposing them for other therapies such as AD [146].

Increased blood pressure has been associated with neurodegenerative disorders, and its lowering, regardless of the therapeutic agent used, may provide neuroprotection. In clinical trials it has been observed that among the RAS modulators, in reducing cognitive decline, ARBs are more effective than ACEIs [147].

There are, however, meta-analyses that argue that the lowering of blood pressure in hypertensive patients may not prevent the risk of cognitive impairment. However, both ACEIs and ARBs could decrease the incidence of AD, independent of their antihypertensive properties [45].

Numerous studies have questioned whether the inhibition of the RAS could be a therapeutic strategy in AD.

In a randomized, double-blind, placebo-controlled trial on a very large number of patients who had suffered stroke or ischemic attack after taking perindopril for 4 years, cognitive decline and dementia were observed to be reduced [25].

On the other hand, ACEIs and ARBs promote the synthesis of Ang-(1-7), an agent with a protective role against vascular senescence, by attenuating oxidative stress, an effect shown in the Mas receptor-deficient mouse model, where a decrease in NO synthesis and an increase in ROS production can be observed [30].

Administration of captopril to aged transgenic mice with AD led to decreased synthesis of beta amyloid plaques [31].

Approved therapies for AD have minimal therapeutic impact and the reduction of amyloid synthesis by various experimental approaches has not been successful. For these reasons, the therapy of neurodegenerative diseases could include the amelioration of major risk factors, such as hypertension, and ARB therapy could be an excellent candidate in brain disorders, due to its beneficial effects [148].

An analysis of data from 30 studies involving more than 850,000 individuals found that those treated with ARBs showed much less cognitive decline than those treated with other antihypertensive drugs or even ACEIs. Another report shows that ACEI therapy given to 784 patients over 3 years was associated with a reduction in the risk of AD [32].

Some renin inhibitors, such as aliskiren, may have beneficial effects in AD because, in in vitro studies, neurotoxicity from Aβ was blocked. These results suggested the need for clinical trials aimed at testing their efficacy in AD [144].

Twenty-five years after the amyloid hypothesis of AD, three Aβ antibodies (aducanumab, crenezumab, and solanezumab) used in clinical trials have suggested a slowing of cognitive decline [149].

## 6. Remarkable Conclusions

In conclusion, it can be affirmed that Ang II elicits a range of cardiovascular effects mediated by the angiotensinergic pathway, including hypertension and increased cerebral irrigation.

The effects of ACEIs (such as captopril, ramipril, lisinopril, etc.) on memory have shown that it is enhanced by the reduction of Ang II synthesis. These effects are not isolated events within a single-dose administration, without interfering memory-disturbing elements. However, they become significant under conditions where their administration coincides with the use of memory-impairing substances like scopolamine. This suggests the presence of mechanisms that exacerbate memory loss caused by Ang and are inhibited by ACE blockers.

The combination of ARBs (such as losartan, candesartan, valsartan, etc.) may exhibit a protective effect when used with pharmacological substances with amnestic effects, such as scopolamine.

Biochemical parameters of oxidative status can be significantly improved by pretreatment with ACE inhibitors, ARBs, or renin inhibitors.

## Figures and Tables

**Table 3:** The effects of telmisartan in various pathological aspects of AD.

Effect on Biological Pathway	Disease Model and Species	Reference
Detected in cultured rat primary cerebellar granule cells exposed to glutamate Absent from human SK-N-SH neuroblasts exposed to IL-1β	In vitro models of neurotoxicity induced by IL-1β and glutamate exposure	[53,54]
Reduced expression of NO, TNF-α, IL-1β activity	In vitro study—BV2 cells stimulated with LPS	[55]
Reduced expression of IL-1β, TNF-α, NF-κB, IL-10 activity	In vitro study—BV2 cells stimulated by soluble Aβ oligomer	[56]
Neuroprotective effects through other mechanisms besides AT1R blockade or PPAR-γ activation Prevention of the increase in LPS-induced inflammatory markers	In vitro study—BV2 cells lacking AT1R expression or PPAR-γ activation	[57]
Reduction of cortical and hippocampal amyloid burden as well as glial activation	In vivo study—5XFAD mice, intranasal administration short-term (1–2 months) and long-term (5 months)	[55,58]
Reduction of Aβ deposition Improvement of cognitive decline induced by Aβ Reduction of the expression of TNF-α and iNOS	In vivo study—ddY mice intracerebroventricular-injected with Aβ1–40	[59,60]
Beneficial effects on learning and memory decline produced by aluminum Reductions in MDA, TNF-α, NF-κβ, Aβ1-42, phosphorylated tau protein levels	In vivo study—rats treated with aluminum chloride	[66]
Neuroprotective effects → Preventing and attenuating cognitive impairment associated with obesity, DM, and acute and chronic stress	In vivo studies	[63,67,68]
Poor penetration of the brain, but enough to block AT1Rs	In vivo study—pharmacokinetic brain PET study in rhesus macaques	[69]
Non-significant difference in cognitive impairment occurring in studied groups	Clinical trials (the ONTARGET and TRANSCEND trials)—high-vascular-risk patients treated with telmisartan, ramipril, a combination of the two, or placebo	[70,71,72]
No change or improved performance, as well as improvement in regional cerebral blood flow in multiple regions of the brain, compared to amlodipine	Clinical trial—the effects of telmisartan vs. amlodipine on cognition and cerebral blood flow in 20 patients with hypertension and probable AD	[73]
Ability to inhibit the short-term decline of glucose metabolism in the anterior olfactory nucleus of the olfactory tract in AD patients’ brains	Clinical trial—FDG-PET study on four hypertensive AD patients regarding brain glucose metabolism	[74]
Lower risk of dementia diagnosis or dementia diagnosis with ischemic stroke as a competing risk and with all-cause mortality as a competing risk	Clinical trial—in hypertensive patients with T2DM	[75]
Synergistic effect between telmisartan and rosuvastatin to reduce the risk of dementia occurrence and the cognitive impairment evolution	Randomized clinical trial—hypertensive patients with/without the APOE ɛ4 allele	[76]
Greater beneficial effects and a reduced incidence of AD in African Americans, but not in non-Hispanic White or European Americans	Clinical trial	[77,78]

Aβ: β-amyloid; AD: Alzheimer’s dementia; AT1R: AT1 receptor; DM: diabetes mellitus; FDG: fluorodeoxyglucose; IL: interleukin; LPS: lipopolysaccharide; MDA: malondialdehyde; NF-κB: nuclear factor-κB; NO: nitric oxide; iNOS: inducible nitric oxide synthase; PET: positron emission tomography; PPAR-γ: proliferator-activated receptor-γ; T2DM: type 2 diabetes mellitus; TNF-α: tumor necrosis factor-α; →: direct involvement.

**Table 4:** The effects of candesartan in various pathological aspects of AD.

Effect on Biological Pathway	Disease Model and Species	Reference
Restoration of the proliferation of neural stem cells previously inhibited by the Aβ25-35 oligomer	In vitro study	[80]
Decrease in the expression of iNOS, NO, COX-2, TNF-α Promotion of the expression of Arg-1 Enhancement of Aβ1-42 uptake by microglia	In vitro study—BV2 cells stimulated with LPS	[81]
Neuroprotective effects → preventing alterations in several transcripts that were up- or downregulated by glutamate	In vitro study—primary neuronal cultures treated with glutamate in excitotoxic concentrations	[82]
Significant decrease in the expression levels of amyloid burden, but only in the hippocampus layer, not in the cortical layers of treated animals	In vivo study—5XFAD mice	[81]
Improvement of spatial memory Decrease in oxidative stress Restoration of acetylcholinesterase activity	In vivo study—mouse model of memory impairment induced by intracerebral administration of streptozotocin	[83,84]
Prevention of neuroinflammation induced by LPS Increase in AT2R expression Prevention of neuroinflammation and astrocyte and microglial activation in the brain	In vivo study—rat model of chronic hypertension	[85,86]
Reduction of neuroinflammation Restoration of function of the endothelial and smooth muscle No significant impact on impaired cognitive function and amyloid plaque load	In vivo study—APP mice	[88]
Reduction of inflammation Restoration of central endothelial function Lack of AT2R inhibition with prevention of neuronal degeneration → Improvement in executive function	Randomized clinical trial—hypertensive patients over 65 years old with early cognitive impairment	[89,90]
Lower brain amyloid deposition Increase in Aβ40 and Aβ42 in CSF No significant modifications on tau protein levels Enhancement of the connectivity in subcortical brain networks	Randomized clinical trial—non-hypertensive adults with prodromal AD	[91]

Aβ: β-amyloid; AD: Alzheimer’s dementia; Arg 1: Arginase-1; AT2R: AT2 receptor; COX-2: cyclooxigenase-2; CSF: cerebrospinal fluid; NO: nitric oxide; iNOS: inducible nitric oxide synthase; LPS: lipopolysaccharide; TNF-α: tumor necrosis factor-α; →: direct involvement.

**Table 5:** The effects of losartan and olmesartan in various pathological aspects of AD.

Compound	Effect on Biological Pathway	Disease Model and Species	Reference
Losartan	Decrease in Aβ plaques, IL-12 p40/p70, IL-1β and GM-CSF Increase in IL-10 → Neuroprotective and anti-inflammatory effects	In vivo study—APP/PS1 transgenic mouse model of AD	[92,93]
	Reduction of Aβ plaques Improvement in neurological deficits and neuroinflammation Increase in choroid plexus cell proliferation, neurogenesis, cell survival, and IL-10 levels in the brain Decreased mortality	In vivo study—rat model of chronic hypertension	[93]
	Prevention of the onset of cognitive dysfunction (learning and memory deficits) and dilatory deficits Soluble Aβ species or plaque load were not decreased Decrease in cerebrovascular and cortical AT1R elevated levels	In vivo study—APP transgenic mice	[96]
	No decrease in the rate of brain atrophy in the studied patients	Randomized placebo-controlled trial (the RADAR trial)—clinically diagnosed mild-to-moderate AD patients (aged 55 years or older)	[97]
Olmesartan	Protective effects in Aβ-induced cellular senescence and neurotoxicity Reduction of ROS, MDA, and senescence biomarkers levels	In vitro study—M17 neuronal cells	[98]
	Attenuation of cognitive decline and improvement of cognitive function Increase in mRNA expression of methyl methanesulfonate sensitive 2 Decrease in superoxide anion production in the brain	In vivo study—high-salt and high-cholesterol diet-fed mice	[99]
	Reduction of oxidative stress in microvessels Attenuation of the cerebrovascular and cognitive impairment	In vivo study—APP23 mice	[100]
	Prevention of vascular dysregulation Improvement of cognitive function	In vivo study—intracerebrovascular Aβ1-40 injected mice	[100]
	Attenuation of cognitive decline Decrease in BBB microvessels permeability Reduction of hippocampal levels of Ang II	In vivo study—Dahl salt-sensitive rats fed with a high-salt diet	[101]
	Prevention of BBB disruption Reduction of oxidative stress Significant improvements in cognitive impairment	In vivo study—5XFAD mice	[102]

Aβ: β-amyloid; AD: Alzheimer’s dementia; AT1R: AT1 receptor; BBB: blood–brain barrier; GM-CSF: granulocyte-macrophage colony-stimulating factor; IL: interleukin; MDA: malondialdehyde; mRNA: messenger ribonucleic acid; ROS: reactive oxygen species; →: direct involvement.

## Data Availability

Not applicable.

## Abbreviations

5-HT	5-hydroxytryptamine
ACE	Angiotensin-converting enzyme
ACEI	Angiotensin-converting enzyme inhibitor
Ach	Acetylcholine
AD	Alzheimer’s dementia
Ang	Angiotensin
APP	Amyloid precursor protein
ARB	Angiotensin II AT1R blocker
Arg-1	Arginase-1
Aβ	β-amyloid
BBB	Blood–brain barrier
CBF	Cerebral blood flow
CNS	Central nervous system
COX-2	Cyclooxigenase-2
CSF	Cerebrospinal fluid
DM	Diabetes mellitus
FDA	Food and Drug Administration
FDG	Fluorodeoxyglucose
GM-CSF	Granulocyte-macrophage colony-stimulating factor
GPCR	G protein-coupled receptor
IL	Interleukin
IRAP	Insulin-regulated aminopeptidase
JNK	C-Jun N-terminal kinase
LPS	Lipopolysaccharide
LTP	Long-term potentiation
MAPK	Mitogen-activated protein kinase
MDA	Malondialdehyde
MLC	Myosin light chain
MLCK	Myosin light chain kinase
NADPH	Nicotinamide adenine dinucleotide phosphate
NF	Nuclear factor
NO	Nitric oxide
iNOS	Inducible nitric oxide synthase
NRTK	Non-receptor tyrosine kinase
PET	Positron emission tomography
PPAR-γ	Peroxisome proliferator-activated receptor-γ
R	Receptor
RAS	Renin–angiotensin system
mRNA	Messenger ribonucleic acid
ROS	Reactive oxygen species
SOD	Superoxide dismutase
T2DM	Type 2 diabetes mellitus
TGF-β1	Transforming growth factor-β1
TNF-α	Tumor necrosis factor-α
